# C-phycocyanin inhibits epithelial-to-mesenchymal transition in Caski cells

**DOI:** 10.1186/s12935-020-01384-8

**Published:** 2020-07-07

**Authors:** Huanhuan Ji, Guoxiang Liu, Jingjing Han, Feng Zhu, Xiaolei Dong, Bing Li

**Affiliations:** Department of Genetics and Cell Biology, Basic Medical College, Qingdao University, 308 Ningxia Road, Qingdao, 266071 People’s Republic of China

**Keywords:** C-phycocyanin, EMT, Caski cells, TGF-β1/smad signal pathway, Cancer

## Abstract

**Background:**

In cervical cancer, most patients die of metastasis. The epithelial-to-mesenchymal transition (EMT) is a pivotal and intricate process that increases the metastatic potential of cervical cancer. C-phycocyanin (C-PC) is a natural marine product isolated and purified from *Spirulina platensis*, has been investigated that has anti-cancer function. The aim of this study was to explore the inhibitory effect of C-phycocyanin on the migration and invasion of cervical cancer cells induced by transforming growth factor-β1 (TGF-β1), so as to provide a new idea for the treatment and prognosis of cervical cancer.

**Methods:**

A wound-healing assay, an invasion assay, immunofluorescence assay, western blot, flow cytometry and real-time reverse transcriptione polymerase chain reaction were explored in cervical cancer Caski cell lines. TGF-β/smad signaling pathway was evaluated of in Caski cell lines.

**Results:**

Our study indicated that TGF-β1 induced EMT in cervical cancer cells. C-phycocyanin inhibited EMT in Caski cells by down-regulating N-cadherin and up-regulating E-cadherin protein expression. Furthermore, C-phycocyanin could inhibit the expression and proteins Twist, Snail and Zeb1 transcription factors related to EMT. In addition, C-phycocyanin could inhibit the migration and invasion of Caski cells induced by TGF-β1. Besides, C-phycocyanin inhibited EMT through TGF-β/smads signaling pathway. We also found C-phycocyanin induced cell cycle G0/G1 arrest by decreasing protein expression levels of Cyclin D1 and p27.

**Conclusions:**

C-phycocyanin reversed TGF-β1-induced epithelial-to-mesenchymal transition in cervical cancer cells and down-regulated the TGF-β/samd signaling pathway induced G0/G1 arrest of tumor cell cycle.

## Background

Cervical cancer is the most common female genital malignant tumor in developing countries. With the extensive development of cervical cancer screening programs [1], early diagnosis and treatment of cervical cancer and precancerous lesions have been improved, but for advanced or metastatic cervical cancer, it has not achieved satisfactory therapeutic effects [2]. Infiltration and metastasis are still the main causes of death in patients with cervical cancer. Studying the underlying mechanism of invasion and metastasis of cervical cancer is particularly important for the treatment and prognosis of cervical cancer [3].

EMT refers to the biological process by which epithelial cells are transformed into mesenchymal phenotype cells by a specific procedure. Under the actions of some factors, epithelial cells lose cell polarity, lose tight connections and adhesion connections between cells, obtain infiltration and migration ability, and become cells with morphology and characteristics of mesenchymal cells [3, 4].There is no doubt that this is also why cancer cells spread in the body. The most familiar change that occurs during EMT is the downregulation of surface E-cadherin expression and upregulation of N-cadherin [5].

TGF-β is an important factor in the induction of EMT in vivo [6]. Previous studies have shown that TGF-β promoted tumor metastasis in genetically engineered mouse models and preclinical studies of TGF-β antagonists showed an anti-tumor effect [7, 8]. The most important signal transducers for the transmission of TGF-β1 signaling are the smads [9]. TGF-β signal requires the binding of TGF-β to TGF-β typeII receptor(TβRII) or the transphosphate of TGF-β typeI receptor(TβRI), and the subsequent phosphorylation of Smad2 and Smad3. Phosphorylation of Smad2/3 binds to Smad4 to form trimer and then translocated to the nucleus [10]. It interacts with transcription factors, co-activating factors and co-inhibitors in the nucleus to inhibit epithelial genes expression but promote the expression of stroma genes. The gene expression mediated by smad 3/4 induced by TGF-β drives the gene reprogramming in the EMT process [11]. Firstly, the expression of the main transcription factors (Snail, Zeb1 and Zeb2) and twist, as well as the synergistic effect of smad 3/4 complex with these transcription factors, are initiated. In addition to Smad signals, TGF-β1 can activate other signaling pathways, such as phosphoinositide 3-kinase (PI3K)–AKT, extracellular signal-regulated kinase (ERK)-mitogenactivated protein kinase (MAPK) and p38MAPK [12].

C-phycocyanin (C-PC) is a natural marine product isolated and purified from *Spirulina platensis*, and it has the characteristics of water solubility and spontaneous red fluorescence [13]. C-phycocyanin has been reported to have good medicinal value, such as improving human immunity, anti-oxidation [14], anti-inflammatory [15] and anti-tumor [13]. More studies have shown that C-phycocyanin exerts anti-cancer effect in various cancer cell types (such as cervical cancer [16], pancreatic cancer [17], non-small cell lung cancer [18] and breast cancer [19, 20] and so on) in vitro and in vivo. However, the effect of C-phycocyanin in EMT of cervical cells is not clear.

In our study, we investigated the effect of C-phycocyanin on TGF-β-induced epithelial mesenchymal transformation in cervical cancer Caski cells, and revealed the molecular mechanism of its anticancer activity. We found that C-phycocyanin inhibited the expression of EMT-related N-cadherin, Twist, Snail and Zeb1, promoted the expression of E-cadherin, and finally inhibited the migration and invasion of Caski cells. In addition, C-phycocyanin triggered G0/G1 phase cell cycle arrest, down-regulating the expression of CyclinD1. In addition, we uncovered that C-phycocyanin mediated EMT inhibition was regulated by inhibiting TGF-β/smad pathway. These results strongly suggested an important role of C-phycocyanin in repressing epithelial–mesenchymal transition and cancer progression.

## Materials and methods

### Reagents and antibodies

C-phycocyanin (lot#R320190412) was purchased from Taizhou Binmei Biotechnology Co., Ltd., Taozhou, China. Recombinant human TGF-β1 (lot#0713AF354-1 L0618) was purchased from Proteintech Co., Ltd., Wuhan, China. Anti-human N-cadherin, E-cadherin, MMP-9, TGF-β ReceptorII, phospho-smad2/3, smad2/3 were purchased from Cell Signaling Technology, Inc. Danvers, MA, USA. The immunofluorescence staining kit (lot No. 13I13H10A0964) was purchased from Boster Biological Engineering Co., Ltd. All other chemicals reached analytical grade.

### Cell culture

Human cervical cancer Caski was provided by the Affiliated Hospital of Qingdao University. Caski was cultured in high glucose DMEM supplemented with 10% (v/v) FBS (Gibco, Thermo Fisher Scientific, Inc.), 100 mg/ml streptomycin and 100 units/ml penicillin in a humidified incubator with 5% CO_2_/95% air atmosphere at 37 °C.

### Western blot analysis

Cells were washed once with ice-cold PBS and lysed in radioimmunoprecipitation assay (RIPA) buffer, after the cell lysates were centrifuged, the protein samples were detected using a BCA protein assay kit (Beyotime Biotechnology, Shanghai, China). Firstly, 30 μg protein was mixed with SDS loading buffer (5×) and heated for 10 min at 95 °C. Then the protein samples were separated by 10% SDS-PAGE, transferred to PVDF membrane (Millipore), the membrane was blocked with 5% bovine serum albumin for 2 h at room temperature and then incubated with specific primary antibodies at 4 °C for overnight. After washed for three times with TBS-Tween 20 (0.1%, v/v), the membrane was incubated with the appropriate HRP-secondary antibody for 1 h. After washed with TBST for three times, the blots were detected using an enhanced chemiluminescence (Millipore).

### Immunofluorescence analysis

Caski cells were treated with 10 ng/ml TGF-β1 and 300 mg/ml C-phycocyanin for 24 h. Subsequently, cells were fixed with 4% paraformaldehyde for 15 min, and blocked with goat serum (1:10) for 30 min, incubated with specific primary antibodies at 4 °C for overnight After washing, cells were exposed to FITC-conjugated goat anti-rabbit second antibody (diluted 1:200). Finally, the nuclei were counterstained with DAPI. Immunofluorescence analysis was detected by ImageXpress^®^ Micro, Molecular Devices (USA).

### Scratch assay

For the Scratch assay, Caski cells (4 × 10 ^5^ cells per well) were plated into 6-well, and cultured until 80–90% confluent. Scratches were created using micropipette tips, then replaced the culture medium with or without 10 ng/ml TGF-β1 and 300 μg/ml C-phycocyanin. The scratches were observed by phase-contrast microscopy and captured at the 0, 24 and 48 h. The percentage of migration was calculated according to the formula:

$$ {\text{Migration }}\left( \% \right) = {\text{Width of the scratch }}\left( {0 - 2 4 {\text{ h}}} \right)/0{\text{ h}} \times 100\% . $$

### Transwell migration and invasion assay

The migration and invasion assay was conducted by using a Transwell chamber with 8.0 mm pore size. In the migration assay, the upper part of the chamber was containing Caski cells (1 × 10^4^ cells/200 μl) with DMEM medium free of FBS. The lower part of the chamber contained 600 μl DMEM with 20% FBS. After incubation for 24 h, washed twice with PBS, chambers were fixed 30 min by 100% methanol, then stained with 0.1% crystal violet for 15 min. Use a cotton swab to erase the upper layer of the cells that are not migrated. In the invasion assay, used a Matrigel-coated Transwell chamber .The steps are consistent with the migration assay. The images were photographed under an EVOS phase-contrast microscope (Thermo Fisher Scientific). Cell number was counted for each field of images and averaged for each chamber.

### Quantitative real-time PCR measurements of mRNA

Total RNA was extracted using TRIzol reagent (Invitrogen) according to the manufacturer’s instruction. Complementary DNAs (cDNAs) were generated using the one-stepiScript cDNA Synthesis Kit (Bio-Rad). The cDNA was synthesized with 1 μg total RNA and random primers by reverse transcription. The mRNA expression levels of genes were detected by real-time quantitative reverse transcription PCR in the ABI 7900HT real-time PCR system. Human glyceraldehyde 3-phosphate dehydrogenase (GAPDH) was normalized to the endogenous reference gene. The levels of N-cadherin, Snail, Twist and GAPDH mRNA were measured by the SYBR Green I assay. N-cadherin was amplified by using the primers with the sequence 5′-GATACTCAGGCAGAGATGATCTACCC-3′ (forward) and 5′-AGACCAGGCACCAGACCAAAGA-3′ (reverse). The Snail primer was 5′-ACCACTATGCCGCGCTCTT-3′ (forward) and 5′-GGTCGTAGGGCTGCTGGAA-3′ (reverse). The Twist primer was 5′-GGCACCATCCTCACACCTCT-3′ (forward) and 5′-GCTGATTGGCACGACCTCT-3′ (reverse).The Zeb1 primer was 5′-AGCAGTGAAAGAGAAGGGAATGC-3′ (forward) and 5′-GGTCCTCTTCAGGTGCCTCAG-3′ (reverse). The GAPDH primer was 5′-ACCCAGAAGACTGTGGATGG-3′ (forward) and 5′-CAGTGAGCTTCCCGTTCAG-3′ (reverse).The reaction was carried out under the following conditions: 95 °C for 5 min, 45 cycles at 95 °C for 5 s, and 60 °C for 30 s. Values of each group mRNA level were calculated as 2^−ΔΔCt^ levels and performed at least four times.

### Flow cytometry to detect cell cycle

The cells were treated with TGF-β and C-phycocyanin for 24 h, and then were digested into cell suspensions with EDTA-free trypsin. The cells were washed 3 times with pre-cooled PBS buffer, and added with 1 ml of pre-cooled 70% ethanol at 4 °C for 2 h followed by adding 25 μl of PI staining solution and incubation at 37 °C for 30 min in the dark. The cell suspension was filtered through a single cell strainer and the cell cycle was detected by flow cytometry.

### Statistical analysis

Each experiment was conducted three or four times independently. The results were expressed by mean ± standard deviation (SD). Statistical analyses were carried out by the Student’s test or the one-way analysis of variance (ANOVA) using a statistical software package (SPSS, USA). P < 0.05 was considered as statistically significant. Statistical significance was also taken as *P < 0.05, and **P < 0.01.

## Results

### TGF-β1 can induce EMT in epithelial cell line Caski

**Fig. 1:**
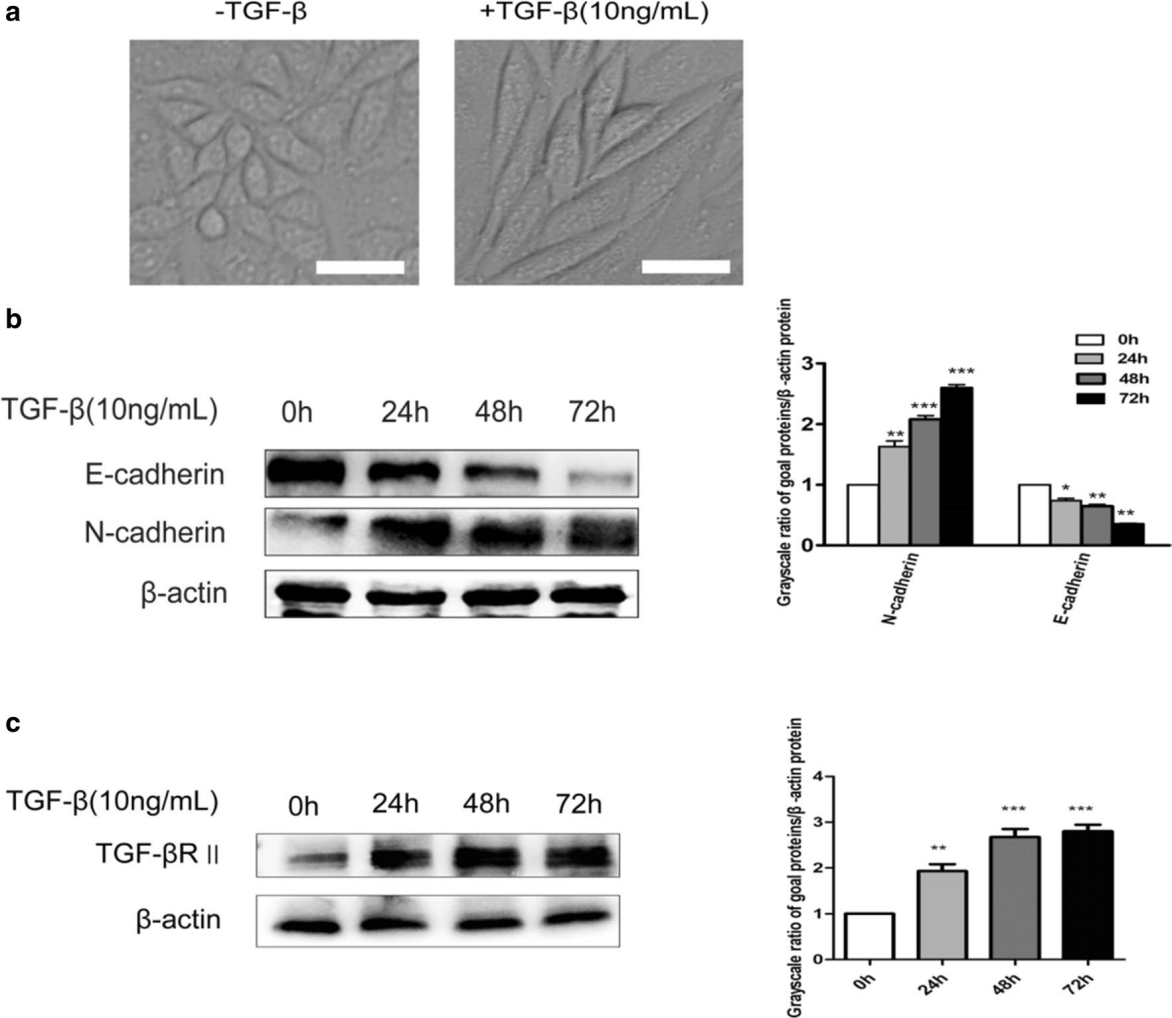
Effects of TGF-β1 on cellular morphological changes and the expressions of E-cadherin, N-cadherin and TβRII in Caski cells. **a** Morphological changes of cells treated with TGF-β1 for 24 h. Scale bar, 10 μm. **b** The expressions of E-cadherin and N-cadherin were determined by western blot. **c** The expressions of TGF-β type II receptor were determined by western blot. Quantitative representation of grayscale ratio of target protein/β-actin. **P < 0.01 vs control group and ***P < 0.001 vs control group

Cervical cancer Caski cells treated with TGF-β1 (10 ng/ml) for 24 h showed a significant change in cell morphology from the original polygon to the long spindle type. The connection between cells and cells became loose, losing the characteristics of the original epithelial phenotype (Fig. 1a). E-cadherin and N-cadherin, the hallmark proteins in the process of EMT [21], were detected in cervical cancer Caski cells after TGF-β1 treatment (0 h, 24 h, 48 h, 72 h) by western blotting. The expression of epithelial phenotype marker protein E-cadherin was gradually decreased, and the expression of the interstitial phenotype marker protein N-cadherin was gradually increased, indicating that TGF-β could induce epithelial–mesenchymal transition in cervical cancer Caski cells (Fig. 1b). Subsequently, we examined the expression of TGF-β type II receptor in the TGF-β signaling pathway. The results showed that the expression of TGF-β type II receptor was positively correlated with the stimulation time of TGF-β1, indicating that TGF-β can affect TGF-β signaling pathway in cervical cancer Caski cells (Fig. 1c). These results indicated that TGF-β could induce epithelial–mesenchymal transition in cervical cancer Caski cells.

### C-phycocyanin can inhibit TGF-β1-induced EMT

**Fig. 2:**
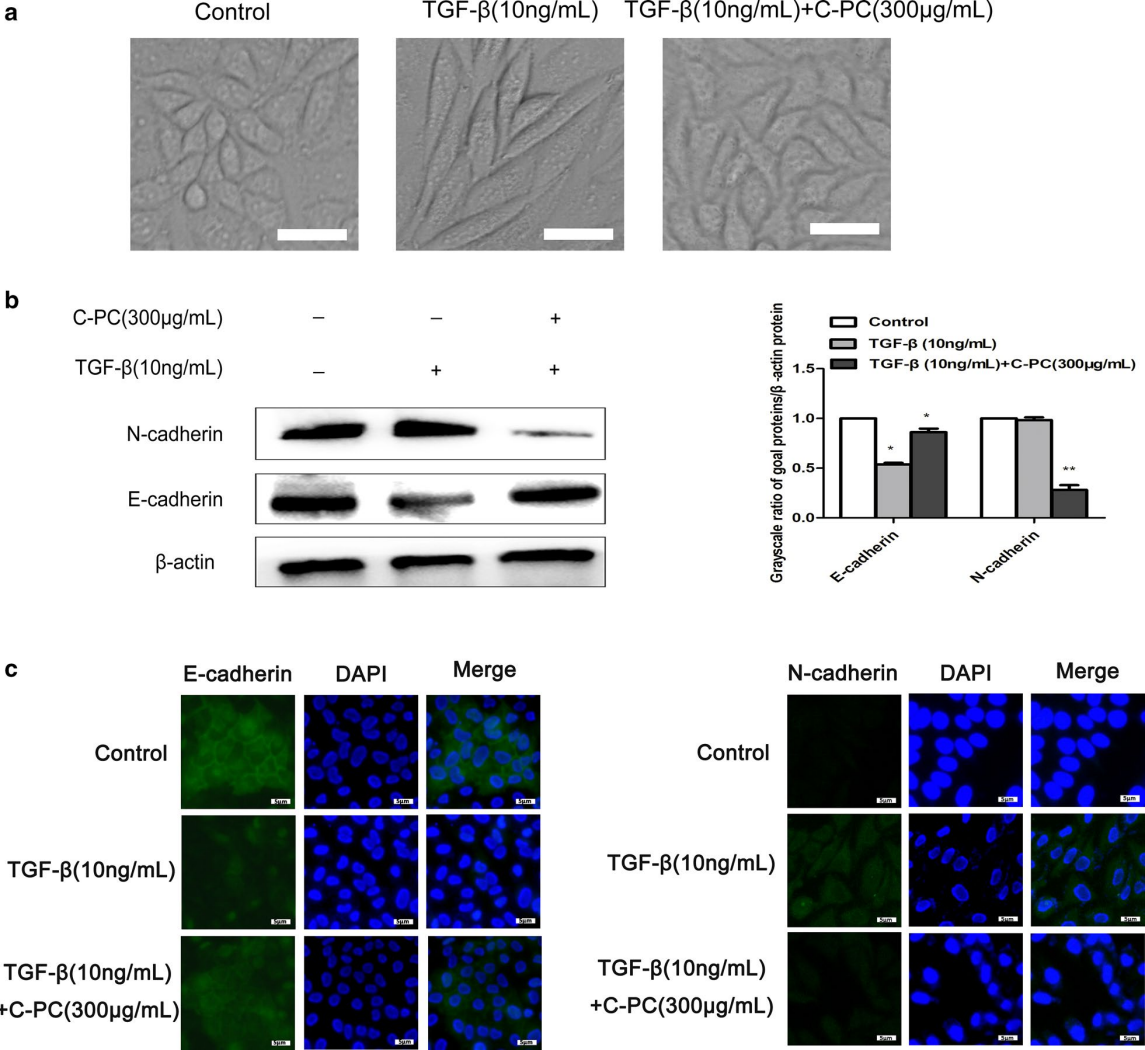
Effects of C-phycocyanin on morphological changes and the expressions of E-cadherin and N-cadherin in Caski cells. **a** Morphological changes of cells treated with TGF-β1 (10 ng/ml) alone, C-phycocyanin (300 µg/ml) alone or both for 24 h. Scale bar, 10 μm. **b** The expressions of E-cadherin and N-cadherin were determined by western blot. Quantitative representation of grayscale ratio of target protein/β-actin. **c** The fluorescence intensity of E-cadherin and N-cadherin detected by immunofluorescence. Scale bars, 5 µm. *P < 0.05, **P < 0.01 vs control group

The cervical cancer Caski cells were treated with TGF-β (10 ng/ml) and treated with C-phycocyanin (300 μg/ml) for 24 h. The morphology of the cells in the combined group was similar to that in the control group, the epithelial phenotype remained normal and the cells were arranged closely (Fig. 2a). Subsequently, we found that C-phycocyanin can increase the level of E-cadherin protein and decrease the protein expression level of N-cadherin by western blot and immunofluorescence (Fig. 2b, c). In Fig. 2b, western blot showed that TGF-β1 upregulated the expression of N-cadherin and down-regulated the expression of E-cadherin and this state was reversed after C-phycocyanin treatment. In Fig. 2c, after TGF-β1 treatment, compared with control group, the fluorescence intensity of E-cadherin decreased but that of N-cadherin increased. However, the fluorescence intensity of E-cadherin increased and the fluorescence intensity of N-cadherin decreased after C-phycocyanin co-treatment. These results indicated that C-phycocyanin was able to inhibit TGF-β-induced epithelial–mesenchymal transition.

### C-phycocyanin can reduce the expression levels of transcription factors Twist, Snail, Zeb1

**Fig. 3:**
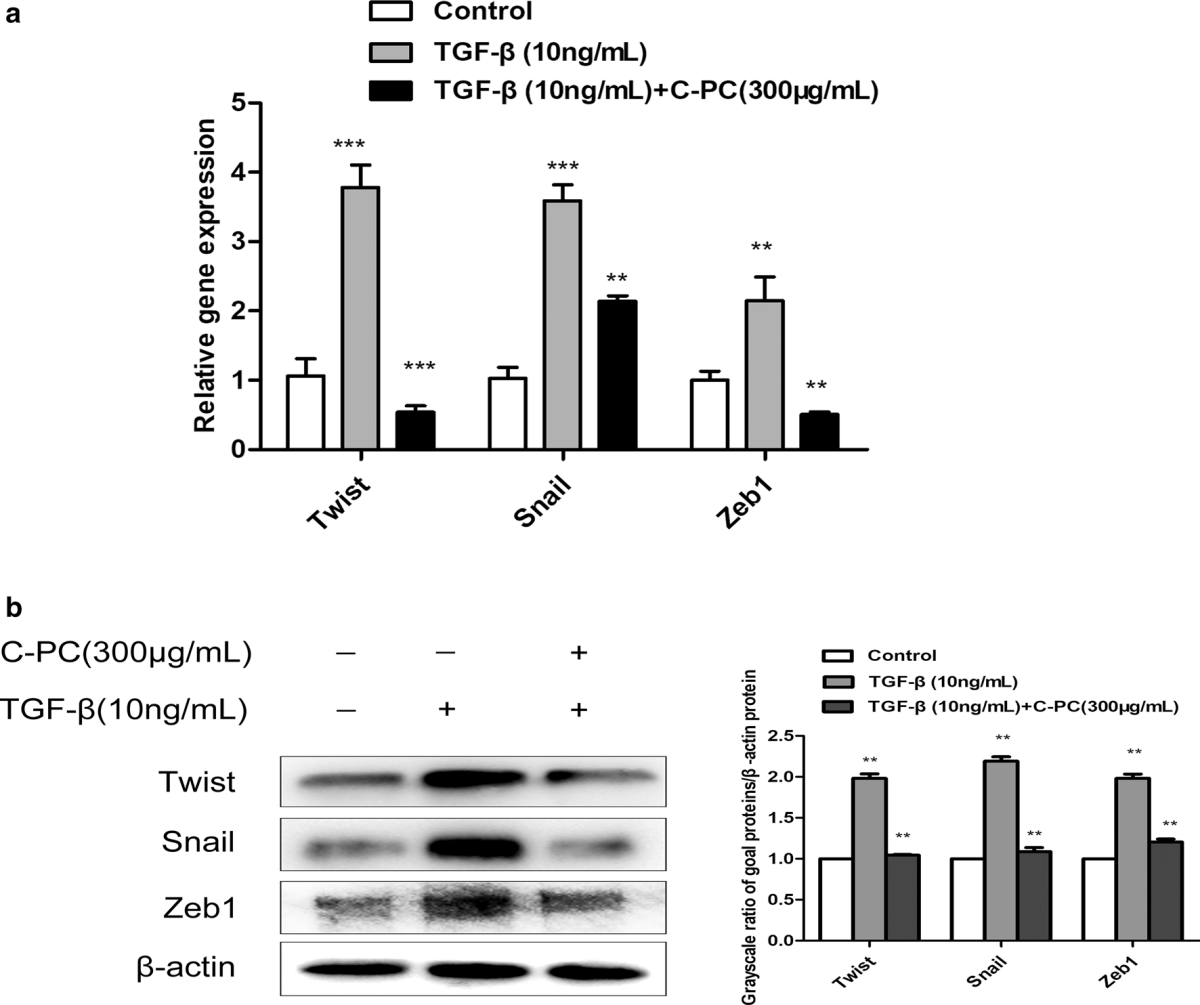
The effect of C-phycocyanin on EMT-related transcription factor Twist, Snail and Zeb1. **a** RT-PCR. The mRNA level of Twist, Snail and Zeb1 in Caski cells treated with TGF-β1 alone and combined with C-phycocyanin was detected. **b** western blot. Twist, Snail and Zeb1 expression levels were detected in cells treatment with TGF-β1 alone and combined with C-phycocyanin. Data are expressed as mean ± SD (n = 3). **P < 0.01, compared with control. ***P < 0.001, compared with only TGF-β1 treated group

To investigate the effect of C-phycocyanin on the mRNA level of EMT-related Twist, Snail and Zeb1, Caski cells were stimulated by TGF-β1 in the presence and absence of C-phycocyanin (300 μg/ml), then evaluated by RT-qPCR. As presented in Fig. 3a, TGF-β1-induced Caski cells, the protein expression and mRNA levels of Twist, Snail and Zeb1 were markedly increased compared with in the control group. However, after 24 h of treatment with TGF-β1 and C-phycocyanin, the expression levels of Twist, Snail and Zeb1 decreased significantly. In addition, we further detected the expression of Snail, Twist and ZEB1 at the protein level. The results showed that TGF-β1 up-regulated the expression of Snail, Twist and ZEB1, and after the addition of C-phycocyanin, the protein expression of Snail, Twist and ZEB1 was down-regulated, as shown in Fig. 3b. In conclusion, these results suggested that C-phycocyanin might inhibit epithelial-to-mesenchymal transition induced by TGF-β1 by reducing the expression of EMT-related transcription factors.

### C-phycocyanin inhibited the TGF-β1-induced migration and invasion of cervical cancer Caski cells

**Fig. 4:**
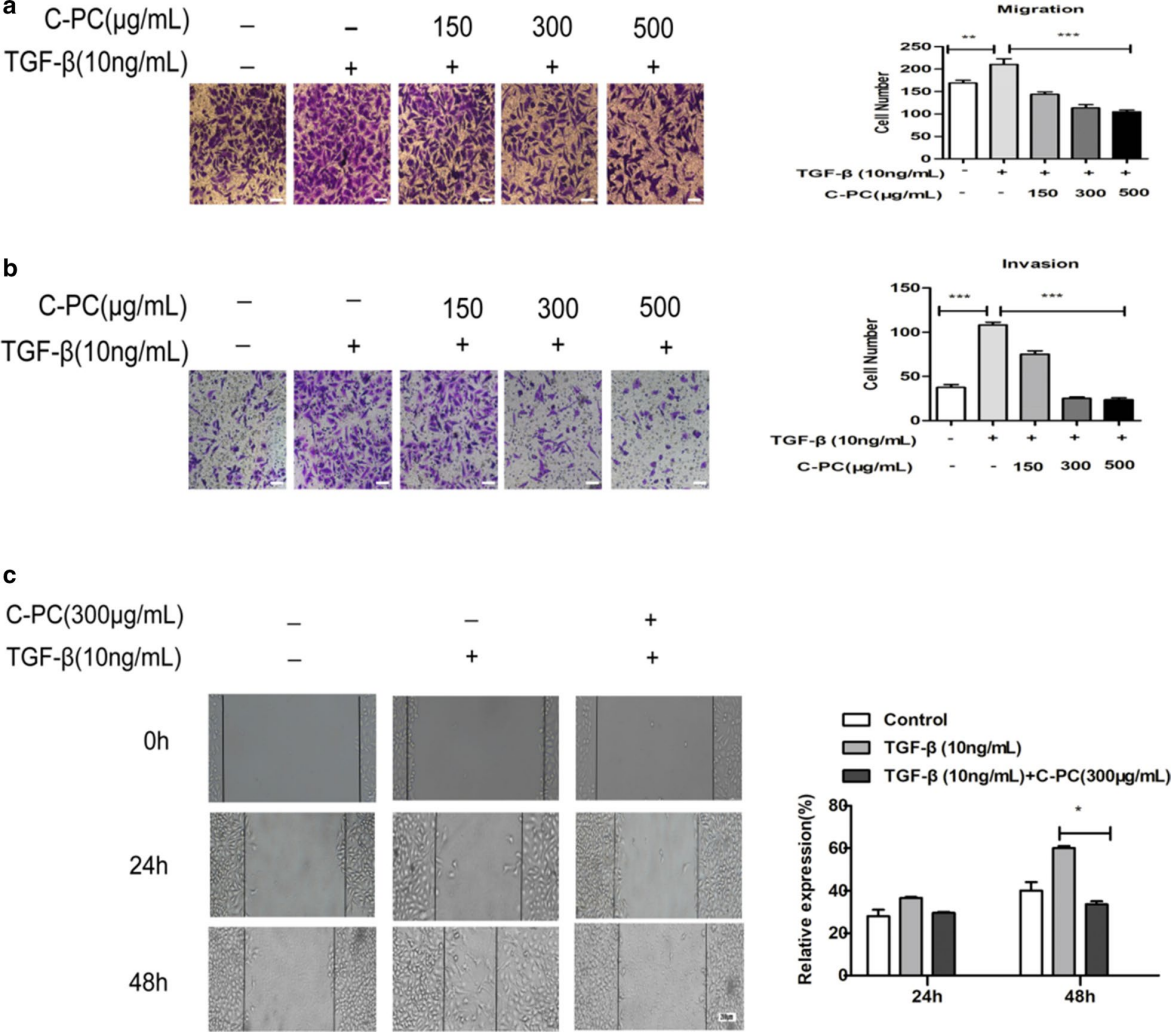
Effect of C-phycocyanin on the TGF-β1-induced cell migration and invasion in Caski cells. **a** Analysis of Caski cells migration by transwell migration assay. Quantitative representation of migration cells after C-phycocyanin treatment for 24 h. Scale bar, 20 μm. **b** Analysis of Caski cells invasion by transwell invasion assay. Quantitative representation of invasion cells after C-phycocyanin treatment for 24 h. Scale bar, 20 μm. **c** Analysis of Caski cells migration by scratch assay. Percentage of cell migration into the wound scratch after C-phycocyanin treatment for 24 h and 48 h. Scale bar, 200 μm. Representative images of wound healing at 0 h, 24 h and 48 h following scratch induction and C-phycocyanin treatment

Recently, some researches had found that tumor cell enhance migration and invasion by TGF-β1 stimulation [6, 8, 21, 22]. To determine the effects of C-phycocyanin on the metastatic abilities, we performed transwell migration, wound-healing, and transwell invasion assays in Caski cells. The results showed that the migration and invasion activity increased significantly after TGF-β1 (10 ng/ml) stimulation in comparison to control cells. In transwell migration assays, different concentrations of C-phycocyanin (150, 300, 500 μg/ml) showed inhibitory effect on migration, and then the inhibitory effect was enhanced with the increase of C-phycocyanin concentration (Fig. 4a). Similarly, in wound scratch migration assays, C-phycocyanin (300 μg/ml) inhibited the migration activity of Caski cells induced by TGF-β1, and showed significant inhibitory effect at 48 h, as shown in Fig. 4b. Furthermore, through transwell invasion assay, we found that C-phycocyanin (150, 300, 500 μg/ml) could inhibit the TGF-β1-induced invasion and the inhibitory effects were in a concentration-dependent manner (Fig. 4c). These results suggested that C-phycocyanin prevented the TGF-β1-induced migration and invasion of Caski cells.

### C-phycocyanin inhibits TGF-β/smad2/3 signaling pathway in Caski cells

**Fig. 5:**
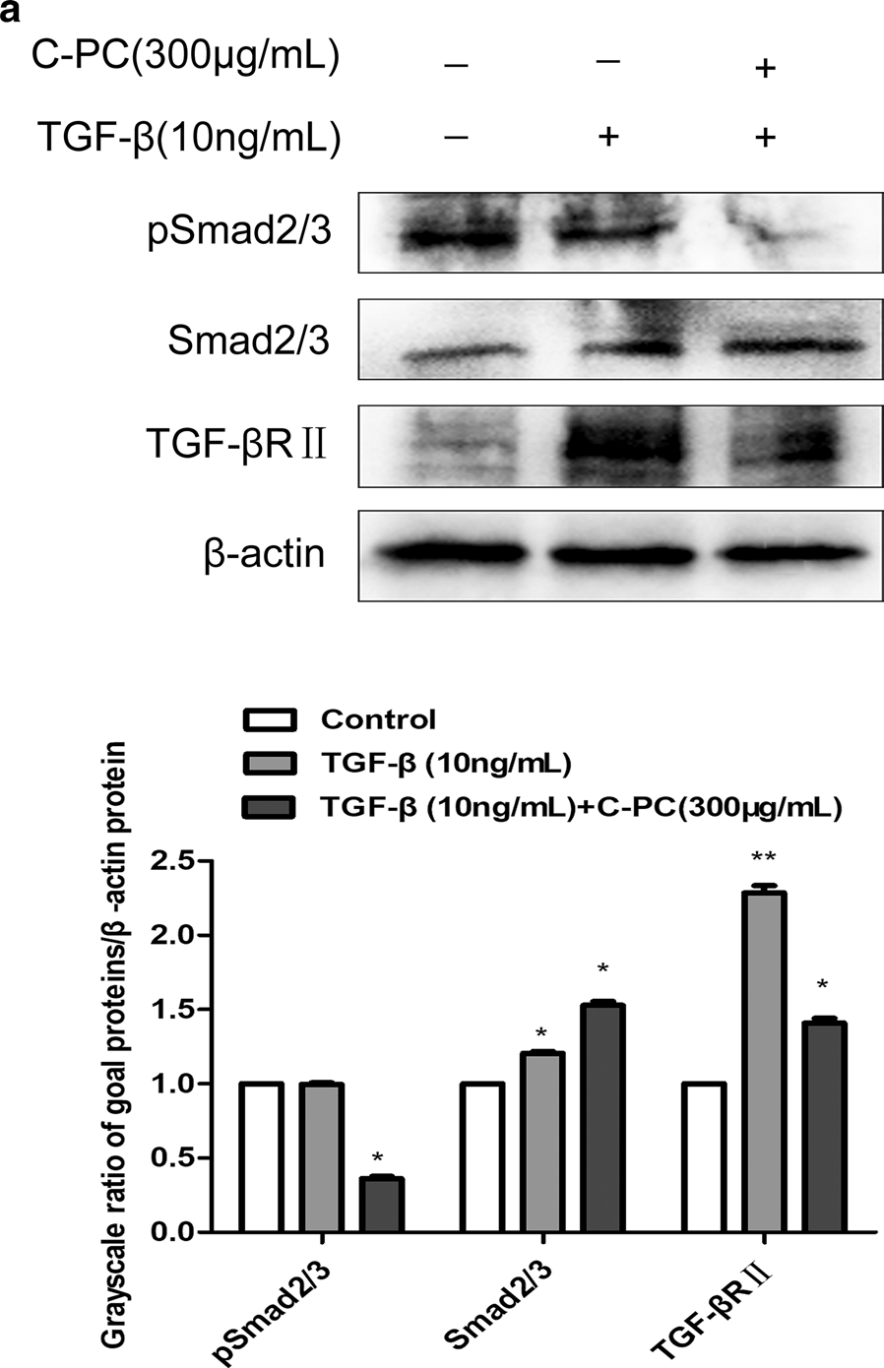
Effect of C-phycocyanin on TGF-β/smad2/3 signaling pathway in Caski cells. **a** After treatments, the expression levels of p-smad2/3 and total smad2/3 were analyzed by western blot. Quantitative representation of grayscale ratio of target protein/β-actin. *P < 0.05, **P < 0.01, compared with control group

There are many kinds of signal pathways that regulate EMT, among which TGF-β/smads signaling pathway plays an important role [21, 23]. In order to study whether C-phycocyanin can inhibit the TGF-β signaling pathway, the protein expression levels of TGF-β typeII receptor and smad2/3 were detected by western blot. The expression of TGF-β typeII receptor was significantly up-regulated after TGF-β1 induction, and down-regulated after C-phycocyanin stimulation. Similarly, the protein expression level of p-Smad2/3 was inhibited with the addition of C-phycocyanin, as shown in Fig. 5a. There was no doubt that these results showed that C-phycocyanin played a certain inhibitory role in TGF- β/samd2/3 signaling pathway.

### C-phycocyan inhibits G0/G1 phase arrest in cell cycle and regulates the proteins expression of p21/p27/CyclinD1 in Caski cells treated with TGF- β1

**Fig. 6:**
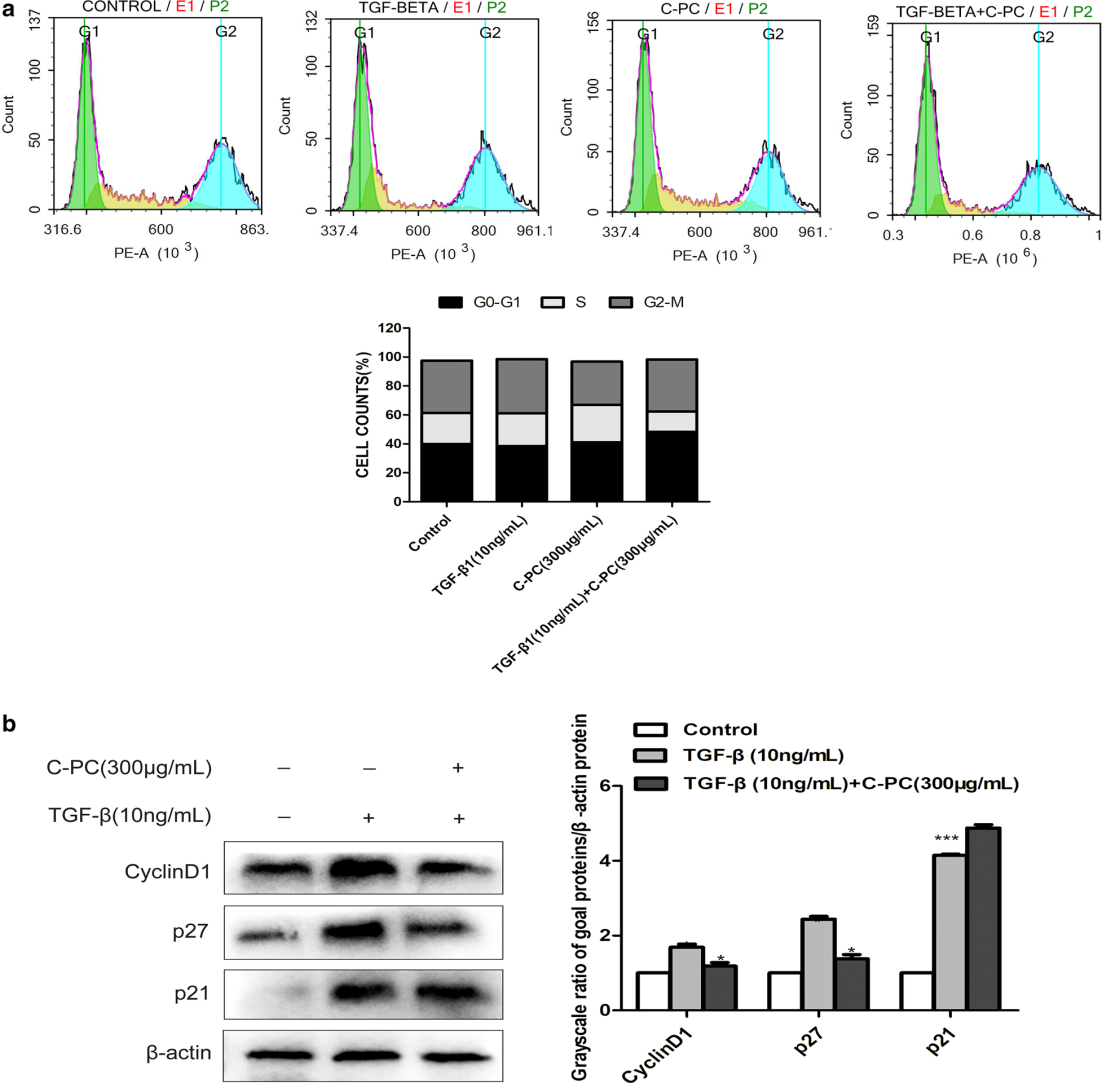
Effects of C-PC on G0/G1 cell cycle arrest in TGF-β-induced Caski cells. **a** C-PC, TGF-β1 and TGF-β1 + C-PC induced G0/G1 cell cycle arrest in Caski cells. Quantitative representation of cell cycle distribution after treatment for 24 h. **b** C-PC, TGF-β1 and TGF-β1 + C-PC induced G0/G1 cell cycle arrest in Caski cells. Quantitative representation of cell cycle distribution after treatment for 48 h

Up to now, our previous studies have shown that C-phycocyanin can inhibit tumor cell cycle arrest [13, 17, 19], but the effect on cancer cells treated with TGF-β is unknown. Therefore, Caski cells were treated with TGF-β1 and C-phycocyanin, and the DNA content of Caski cells was detected by flow cytometry. After 24 h of treatment, the proportion of G0/G1 phase of Caski cells treated with control group, TGF-β1 alone group, C-phycocyanin alone group and combination group was 39.96%, 38.52%, 41.10% and 48.26%, respectively, and the proportion of S phase was 21.26%. 22.58%, 25.89% and 14.12%, and the proportion of G2 phase was 36.29%, 37.46%, 29.90% and 35.99% (Fig. 6a). TGF- β alone could decrease the number of cells in G0/G1 phase, and increase of cell number in G2/M phase. C-phycocyanin alone could induce G0/G1 cell cycle arrest in Caski cells. The inhibitory effect of C-phycocyanin on the cells treated with TGF- β1 was more significant. In order to further study the mechanism of C-phycocyanin and TGF- β inhibiting cell cycle, we detected the levels of cell cycle regulatory proteins involved in G1/S conversion, including CyclinD1, CDK inhibitors p21 and p27. We found that the protein expression levels of CyclinD1, p21 and p27 increased after 24 h of treatment with TGF- β1. At the same time, C-phycocyanin decreased the protein expression of CyclinD1 in Caski cells treated with TGF- β1. The expression level of p21 increased slightly, on the contrary, the protein expression level of p27 decreased (Fig. 6b). In addition, it has been confirmed that Cyclin D1 and p27 play an important role in promoting fibroblasts migration. All these results suggested that C-phycocyanin could inhibit the expression of CyclinD1 and p27 in Caski cells treated with TGF- β1.

## Discussion

Cervical cancer is one of the most common malignant tumors in women. Most of the histological types of cervical cancer are squamous cell carcinoma [24]. According to reports, the vast majority of countries in the world overall cervical cancer ranked second in cervical cancer, but in sub-Saharan Africa and most of South Asia, the five-year prevalence rate ranked first [1]. More than 500,000 new cases of cervical cancer are confirmed each year, with a related death toll of about 250,000 [25]. At present, the treatment of cervical cancer is mainly surgery combined with radiotherapy, but the clinical symptoms of cervical cancer are not obvious at the initial stage of the disease. Sometimes, the cancer has already metastasized and cannot be treated by surgery. The speciality of radiotherapy is poor. As a result, the mortality rate of patients remains high. It has been found that the invasion and metastasis of epithelial malignant tumors are mostly related to the acquisition of EMT characteristics, because the basis of tumor invasion is the decrease of tumor cell adhesion and the enhancement of migration and motility [4, 8]. Therefore, the inhibition of early metastasis of tumor, to clarify the molecular mechanism of tumor metastasis, to find effective measures to control its metastasis is the focus of cervical adenocarcinoma research. It has been reported that C-phycocyanin can inhibit the proliferation of many kinds of tumor cells, induce tumor cell cycle arrest, and promote tumor cell apoptosis and autophagy. However, at present, there are few detailed reports on the effect of C-phycocyanin on EMT.

In the early stage of tumorigenesis, TGF-β can inhibit cell proliferation and induce apoptosis, but in the advanced stage of tumor, it can promote tumor cell infiltration and metastasis by inducing angiogenesis and promoting immune escape [6]. It is reported that TGF β1 can induce culture in vitro during the development of some epithelial cell lines and embryos, such as TGF-β promoting the culture of pancreatic cancer Panc-1 cells in vitro [26]. In this study, Caski cells treated with TGF-β showed interstitial characteristics, such as spindle cell-like morphology, down-regulation of E-cadherin, up-regulation of N-cadherin and TGF-β type II receptor, while cells treated with C-PC returned to epithelial phenotype.

Metastasis is a multicellular process involving the adhesion, migration and invasion of cancer cells [27]. A previous study has reported that Snail is a transcriptional regulator and upstream regulator of E-cadherin, which negatively regulates the expression of E-cadherin. When EMT occurs, Snail is up-regulated and E-cadherin is down-regulated, which promotes the transformation of epithelial cells to mesenchymal phenotype, thus promoting tumor metastasis [3]. Zeb1 is an important component of the Zeb transcription factor family. The expression level of Zeb1 is negatively correlated with the expression level of E-cadherin. Zeb1 binds to the E-box sequence of E-cadherin and suppresses the transcription of E-cadherin [28]. Twist is an important transcription factor in EMT, which not only plays an important role in development. At the same time, it also has the ability to promote tumor invasion and metastasis, and has a high level of expression in a variety of malignant tumors [29]. In our study, the mRNA expression level of Twist, Snail, Zeb1 was significantly up-regulated in the cells treated with TGF-β1, but down-regulated by C-phycocyanin. These results suggested that C-phycocyanin may inhibit the EMT of Caski cells by down-regulating the expression of transcription factors. In addition, we further found that C-phycocyanin prevented the TGF-β1-induced migration and invasion of Caski cells.

The most important signal transducers for TGF-β signaling are the Smads. The Smad protein family can be divided into three groups: receptor-activated Smads (R-Smads), universal ligand-type Smads (Co-Smads), and suppressive Smads (I-Smads) [30]. In the TGF-β signaling pathway, TGF-β family members bind to the serine/threonine kinase receptor to form and phosphorylate the heterotetrameric complex TGF-β type II receptor (TβRII), which in turn activates TGF-β1 receptor (TβRI). TβRI phosphorylates downstream R-Smads (Smad2 and Smad3), and R-Smads binds to Co-smad (smad4) to form a complex, enter cells, and accumulate in the nucleus [31]. This complex, together with other transcription factors (transcription factors, TFs), binds to the DNA duplex and regulates specific gene transcription [32]. In this study, our results showed that C-phycocyanin could effectively reduce the protein expression of TGF-β type II receptor and pSmad2/3, and then inhibit the TGF- β signaling pathway in cervical cancer Caski cells.

Cyclin D1 is one of the members of the D-type cell cycle protein family that regulates the G1/S phase transition of the cell cycle [33]. It is reported that cyclinD1 binding proteins p21 and p27 also regulate migration. P21 has an inhibitory effect on the number of genes related to controlling S phase and mitosis [34]. The enhanced expression of p21 leads to cell cycle arrest, which often occurs during G2/M transition and is accompanied by polyploidy [35]. P27 is an unstructured multifunctional protein that affects a variety of biological processes from cell cycle regulation to cell migration and transcriptional regulation [36]. Notably, it has been reported that in addition to the loss of nuclear p27, the increase of cytoplasmic p27 in many human tumors is usually related to the invasiveness of tumors and the poor prognosis of patients [37, 38]. Our results showed that the expression of p21 in Caski cells treated with TGF-β was significantly increased, which may be related to the inhibition of cell cycle arrest in G2/M phase. The expression of cyclinD1 but p27 was increased protein down-regulated after C-PC treatment, which might be related to the inhibition of cell cycle arrest in G0/G1 phase as well as the inhibition of cell migration.

## Conclusions

The study demonstrated that TGF-β1 can induce epithelial-to-mesenchymal transition in cervical cancer cells, and C-phycocyanin can reduce the invasion and migration of cervical cancer cells. Down-regulation of TGF-β/samd2/3 signal pathway can prevent epithelial–mesenchymal transformation and induce G0/G1 arrest of tumor cell cycle. These results suggest that C-phycocyanin can be used as a promising anticancer drug on cervical cancer.

## Data Availability

The authors declare that the data supporting the findings of this study are available within the article

## Abbreviations

C-PC: C-phycocyanin
EMT: epithelial-to-mesenchymal transition
TGF-β1: transforming growth factor-β1
TβRI: TGF-β typeI receptor
TβRII: TGF-β typeII receptor
